# Heat shock protein 90 is an essential molecular chaperone for CB2 cannabinoid receptor–mediated signaling in trabecular meshwork cells

**Published:** 2012-11-29

**Authors:** Fang He, Akhilesh Kumar, Zhao-Hui Song

**Affiliations:** Department of Pharmacology and Toxicology, University of Louisville School of Medicine, Louisville, KY

## Abstract

**Purpose:**

To examine the interaction of heat shock protein 90 (Hsp90) with the CB2 cannabinoid receptor in trabecular meshwork (TM) cells and to investigate the roles of Hsp90 in CB2 receptor–mediated cell signaling and actin cytoskeleton remodeling.

**Methods:**

Coimmunoprecipitation experiments and western blot analyses, using specific anti-CB2 and anti-Hsp90 antibodies, were performed to study the interaction of Hsp90 with the CB2 receptor in TM cells. An antiphospho-extracellular-signal-regulated kinases 1/2 (ERK1/2) antibody was used to detect the CB2 receptor–mediated phosphrylation of ERK1/2. In cytoskeleton studies, Alexa Fluor 488–labeled phalloidin staining was used to examine actin filaments of TM cells. PD98059, a specific inhibitor of the ERK1/2 pathway, was used to evaluate the role ERK1/2 pathway in CB2 receptor–mediated actin cytoskeleton changes. Geldanamycin, an inhibitor of Hsp90, was used to investigate the roles of Hsp90 in CB2 receptor–mediated ERK1/2 phosphorylation and actin cytoskeleton remodeling.

**Results:**

The interaction of Hsp90 with the CB2 receptor was established in TM cells with coimmunoprecipitation experiments and western blot analyses. Treatment of TM cells with geldanamycin significantly inhibited the interaction of Hsp90 with the CB2 receptor. Disruption of the CB2/Hsp90 interaction by treating TM cells with geldanamycin inhibited CB2 receptor–mediated ERK1/2 phosphorylation, as well as actin cytoskeleton remodeling. Furthermore, treatment of TM cells with PD98059 profoundly attenuated CB2 receptor–mediated actin cytoskeleton changes.

**Conclusions:**

The data from this study establish a specific interaction between Hsp90 and the CB2 receptor in TM cells. In addition, the current study demonstrates that by interacting with the CB2 receptor, Hsp90 plays an important role as a molecular chaperone in CB2 receptor–mediated cell signaling and actin cytoskeleton rearrangement in TM cells.

## Introduction

Glaucoma is one of the leading causes of blindness in the world, and elevated intraocular pressure (IOP) is one of the major risk factors for glaucoma [1–3]. Currently, IOP-lowering drugs are used as frontline treatments for glaucoma. However, new and better therapeutic agents for glaucoma with reduced side effects and enhanced therapeutic properties are highly desirable. Marijuana smoking was first reported to reduce intraocular pressure (IOP) in 1971 [4]. Following this initial observation, many studies conducted on human subjects and animal models have confirmed the IOP-lowering properties of marijuana and cannabinoids [5–10]. Due to their IOP-lowering properties, cannabinoids have been proposed as a new class of antiglaucoma drugs. However, a major problem of cannabinoids as therapeutic agents is their severe psychoactive effect. The cloning of cannabinoid receptor subtypes and the development of subtype-selective cannabinoid ligands have provided us with new hope for better separation of the therapeutic effects of cannabinoids from their undesired psychoactive side effects. Two G protein-coupled coupled receptors (GPCRs), CB1 and CB2 cannabinoid receptors, are established targets of cannabinoid ligands [11–13]. CB1 receptors are located in the central nervous system (CNS) as well as in the periphery, whereas CB2 receptors are mainly located in the peripheral tissues [11–13]. Because of the lack of distribution of CB2 receptors in the brain, cannabinoid drugs that act specifically on CB2 receptors should be devoid of the psychoactive effects of marijuana.

Maintaining IOP depends on a dynamic balance between the secretion of aqueous humor by the ciliary body and the outflow of aqueous humor through the trabecular meshwork (TM) and uveoscleral route [1]. The TM is a major site for aqueous humor outflow and thus is important for regulating IOP [1]. Previously, we found that functional CB2 receptors are expressed in TM cells [14,15]. More importantly, we discovered that the TM CB2 receptor is involved in cannabinoid-induced enhancement of aqueous humor outflow facility [14]. This suggests that CB2 cannabinoid receptors in the eye may be explored as a therapeutic target for developing non-psychoactive IOP-lowering cannabinoids. Therefore, understanding the molecular mechanisms underlying the CB2 receptor–mediated enhancement of aqueous humor outflow is particularly important.

Recent evidence has strongly suggested that GPCRs interact with a wide variety of proteins in addition to G proteins, which can participate in the trafficking, signaling, fine-tuning, and allosteric regulation of GPCRs [16]. Heat shock protein 90 (Hsp90) is a highly conserved protein. Its role as a molecular chaperone in preventing protein aggregation and in promoting refolding of denatured proteins has been well established [17–19]. More recently, interest in Hsp90 has expanded to include an apparently mechanistically distinct function. By forming heterocomplexes with its substrates, Hsp90 has been shown to play a key role as a scaffolding site for various signaling events under non-stress conditions [17–19]. Previously, we revealed that there are specific interactions between Hsp90 and CB2 receptors in HEK293 cells expressing transfected CB2 receptors as well in differentiated HL-60 cells expressing endogenous CB2 receptors [20]. Furthermore, we have demonstrated that Hsp90 is involved in the CB2 receptor signaling and CB2 receptor–mediated cell migration in CB2-transfected HEK293 cells and HL-60 cells [20].

The state of the TM cell actin cytoskeleton is an important determinant of aqueous fluid outflow through the TM [21]. Previously, we reported that CB2 agonists induce changes in TM cell morphology and rearrangements of TM cell actin cytoskeleton [15]. To better understand the mechanisms of CB2 agonist–induced enhancement of aqueous humor outflow facility, in this study we tested the hypothesis that in TM cells, Hsp90 might interact with the CB2 receptor and play an important role in CB2 receptor–mediated cell signaling and actin cytoskeleton changes.

## Methods

### Materials

Penicillin/Streptomycin mixture and Dulbecco’s Modification of Eagle’s Medium (DMEM) were purchased from Fisher Scientific (Pittsburg, PA). JWH 015 was purchased from Tocris (Baldwin, MO). SR144528 was obtained from the National Institute of Drug Abuse (Rockville, MD). Geldanamycin was from BIOMOL Research Laboratories (Plymouth Meeting, PA). PD98059, fibronectin, and fatty acid-free BSA (BSA) were bought from Sigma-Aldrich (St. Louis, MO). Alexa Fluor 488-conjugated phalloidin was purchased from Molecular Probes, Inc. (Eugene, OR). Rabbit polyclonal anti-CB2 antibody was purchased from Cayman Chemicals (Ann Arbor, MI). Mouse monoclonal anti-Hsp90 antibody was purchased from BD Bioscience (San Jose, CA). Rabbit anti-mouse immunoglobulin G was purchased from Chemicon (Temecula, CA). His-Tag monoclonal antibody was from EMD Bioscience (Madison, WI). Polyclonal anti-ERK1/2 antibody and monoclonal antiphospho-ERK1/2 (Thr202/Tyr204) antibody were purchased from Cell Signaling Technology (Beverly, MA). Horseradish peroxidase–conjugated goat anti-rabbit secondary antibody and anti-mouse secondary antibody, and enhanced chemiluminescence (ECL) kit were purchased from GE Healthcare (Piscataway, NJ).

### Trabecular meshwork cell culture

The TM was isolated from fresh porcine eyes by blunt dissection. Culture of TM cells was performed according to previously published methods [22,23]. Explants of dissected trabecular tissue were placed in Dulbecco's modified Eagle's medium (DMEM) supplemented with 10% (v/v) fetal bovine serum, 100 U/ml penicillin, 100 mg/ml streptomycin, and 20 mM L-glutamine, and incubated at 37° under a 10% CO_2_ atmosphere. The tissue explants were left undisturbed for 2 weeks. During this period, they attached to the culture dish, and cells migrated spontaneously from all areas of the explants. After 2 weeks of plating, the tissue explants were removed, and the cells were passed. The identity of TM cells was established by their morphology and their ability to take up acetylated low-density lipoprotein and to secrete tissue plasminogen activator.

### Membrane preparation and solubilization

For membrane preparations, cells were washed twice with cold phosphate-buffered saline (PBS; 137 mM NaCl, 2.7 mM KCl, 10 mM Na_2_HPO_4_•2H_2_O, 2 mM KH_2_PO_4_, pH 7.4), scraped off the tissue culture plates, and collected by centrifugation at 1000 × *g* for 5 min at 4 °C. Subsequently, the cells were homogenized in membrane buffer (50 mM Tris–HCl, 5 mM MgCl_2_, and 2.5 mM EDTA, pH 7.4) with a Polytron homogenizer (Kinematica, Basel, Switzerland). After the homogenate was centrifuged at 32,000 × *g* for 20 min at 4 °C, the pellet was resuspended in membrane buffer containing 1% dodecyl maltoside and the sample was incubated with rotation for 2 h at 4 °C. The solubilized samples were then centrifuged for 20 min at 100,000 × *g* for 20 min at 4 °C, and the protein content in the supernatant was determined using a Broadford protein assay kit from Bio-Rad (Hercules, CA).

### Immunoprecipitation

For coimmunoprecipitation experiments, 500 μg proteins from solubilized membrane preparations were incubated with 20 μg primary antibody and 80 μl protein G-Sepharose beads. After overnight incubation at 4 °C, the resins were washed with 10 volumes of solubilization buffer with increasing concentrations of NaCl, and proteins were eluted from beads in sample loading buffer.

### Western blot analysis

Samples were incubated with 2X Laemmli buffer under reducing conditions at room temperature for 20 min, and proteins were resolved on a 10% sodium dodecyl sulfate (SDS)–polyacrylamide gel using an Invitrogen Mini Gel electrophoresis system (Grand Island, NY). Proteins were transferred onto a nitrocellulose membrane for immunodetection with the indicated antibodies. The nitrocellulose membranes were blocked with 5% nonfat dry milk in Tris-buffered saline/Tween-20 (TBS-T; 20 mM Tris, 137 mM NaCl, and 0.1% Tween-20, pH 7.6) for 1 h and then incubated overnight at 4 °C with the primary antibody. Thereafter, the membranes were washed twice for 10 min each time with TBS-T buffer and incubated with horseradish peroxidase-conjugated secondary antibody for 1 h at room temperature. The membranes were then washed three times with TBS-T buffer for 10 min each time, and the antibody-recognized protein bands were visualized using an enhanced chemiluminescence kit from GE Healthcare (Piscataway, NJ).

### **Extracellular-signal-regulated kinases 1/2** (**ERK1/2) phosphorylation assay**

The TM cells were seeded into six-well plates at a density of 2 × 10^5^ cells per well and were grown to confluence. TM cells were maintained in serum-free medium overnight, and then the TM cells were treated with drugs for 15 min. For antagonism experiments, the cells were pretreated with vehicle, antagonist, or inhibitor for 15 min. The cells were then treated with agonist for 15 min. At the end of the treatment period, cells were washed with ice-cold PBS, and 100 µl of ice-cold lysis buffer containing 50 mM β-glycerophosphate, 20 mM EGTA, 15 mM MgCl_2_, 1 mM NaVO_4_, 1 mM dithiothreitol (DTT), 1 mM phenylmethylsulfonyl fluoride, and 1 µg/ml of a protease inhibitor cocktail (Roche Diagnostics, Indianapolis, IN) were added. The whole cell lysate was clarified with centrifugation at 14,000 × *g* for 10 min, the supernatants were collected, and total protein concentration was measured using the Bradford protein assay reagent (Bio-Rad, Hercules, CA). About 50 µg of proteins were mixed with 4X Laemmli sample buffer, and after boiling for 5 min, proteins were separated on a 10% sodium dodecyl sulfate–polyacrylamide gel. Subsequently, the proteins were transferred onto a nitrocellulose membrane, and the membranes were blocked with 3% non-fat milk. The blots were probed with a monoclonal antiphospho-ERK1/2(Thr202/Tyr204) antibody. Antibody binding was visualized with enhanced chemiluminescence western blotting detection reagents (GE Healthcare, Piscataway, NJ). The membranes were then stripped and reprobed for total ERK1/2 using a rabbit polyclonal anti-ERK1/2 antibody.

### Phalloidin staining of actin cytoskeleton

TM cells were allowed to grow to confluence on sterile glass coverslips precoated with 5 μg/ml fibronectin and were starved in DMEM for 48 h. TM cells were then treated with JWH 015 for 3 h. Subsequently, cells were washed twice with PBS, fixed with freshly prepared 4% paraformaldehyde for 15 min, washed twice with PBS, permeabilized with 0.5% Triton-X100/PBS for 10 min, washed twice again with PBS, and then blocked for 1 h with 1% BSA/PBS. For actin cytoskeleton staining, Alexa Fluor 488–conjugated phalloidin was added onto permeabilized and BSA-blocked cells at a concentration of 0.7 units/ml for 1 h. Finally, coverslips were mounted with Vectashield (Vector Laboratories, Burlingame, CA) and viewed with a fluorescence microscope (model IX50; Olympus, Lake Success, NY).

### Data analysis

For ERK1/2 phosphorylation assay, the bands on X-ray films were scanned (Personal Densitometer SI; Molecular Dynamics, Sunnyvale, CA) and were quantified using the ImageQuant software (Molecular Dynamics). The bar graphs were generated with GraphPad Prism software (La Jolla, CA), and the data were expressed as mean±standard error of the mean (SEM). One-way ANOVA (ANOVA) with the Newman–Keuls post-test was used to compare the data points of the different treatment groups. The level of significance was chosen as p<0.05.

## Results

### Identification of heat shock protein 90 as a CB2 receptor interacting protein in trabecular meshwork cells

**Figure 1:**
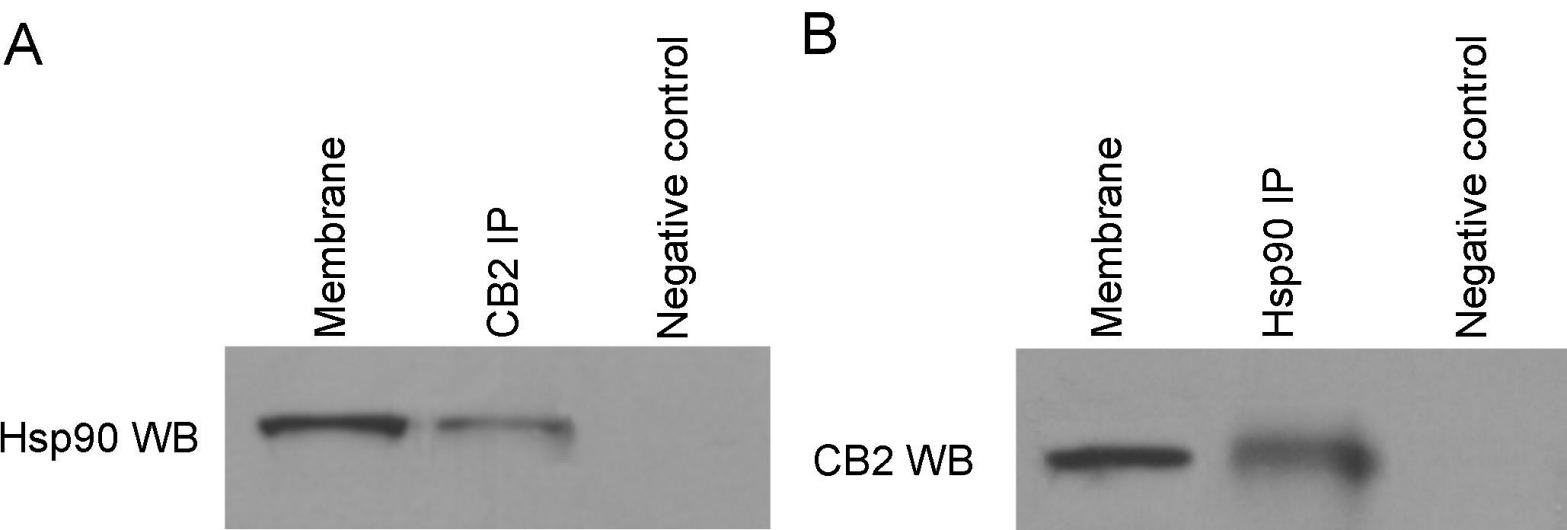
Identification of Hsp90 as a CB2 receptor interacting protein in trabecular meshwork cells. **A**: Membranes prepared from trabecular meshwork (TM) cells were subject to immunoprecipitation with the anti-CB2 antibody, and were then immunoblotted with the anti-Hsp90 antibody. **B**: Membranes prepared from TM cells were subject to immunoprecipitation with the anti-Hsp90, and were then immunoblotted with the anti-CB2 antibody. Control experiments were performed using non-relevant IgGs. The experiment was repeated three times, with similar results. Hsp90 was coimmunoprecipitated by the anti-CB2 antibody, whereas the CB2 receptor was coimmunoprecipitated by the anti-Hsp90 antibody from solubilized TM cell membrane preparations.

As the first step to test the hypothesis that Hsp90 is essential for CB2-mediated signaling to actin cytoskeleton changes in TM cells, we identified the interactions of Hsp90 with the CB2 receptor in TM cells with coimmunoprecipitation experiments and western blot analyses. The CB2 receptor protein complex was immunoprecipitated by using an anti-CB2 antibody and then immunoblotted with an anti-Hsp90 antibody. To ensure the specificity of the results, equal amounts of solubilized membrane proteins from TM cells were incubated with a non-relevant antibody as negative control. As shown in Figure 1A, the Hsp90 band was detected in TM cell membrane preparations, and Hsp90 was coimmunoprecipitated with the CB2 receptor in solubilized TM cell membrane preparation, whereas the Hsp90 signal was absent when membrane proteins were incubated with the non-relevant antibody.

To further confirm the interactions of Hsp90 with the CB2 receptor, coimmunoprecipitation and immunoblotting experiments were performed in reverse order, i.e., anti-Hsp90 was used for immunoprecipitation to see if the CB2 receptor could be coimmunoprecipitated. As shown in Figure 1B, the CB2 band was detected in TM cell membrane preparations, and the CB2 receptor was coimmunoprecipitated with Hsp90 from the solubilized membrane preparations. However, the CB2 band was not detected when the solubilized membrane preparations were incubated with a non-relevant antibody. These data clearly demonstrated the specific interactions between Hsp90 and the CB2 receptor in TM cells expressing the endogenous CB2 receptor.

### The effect of geldanamycin on the interaction between heat shock protein 90 and the CB2 receptor in trabecular meshwork cells

**Figure 2:**
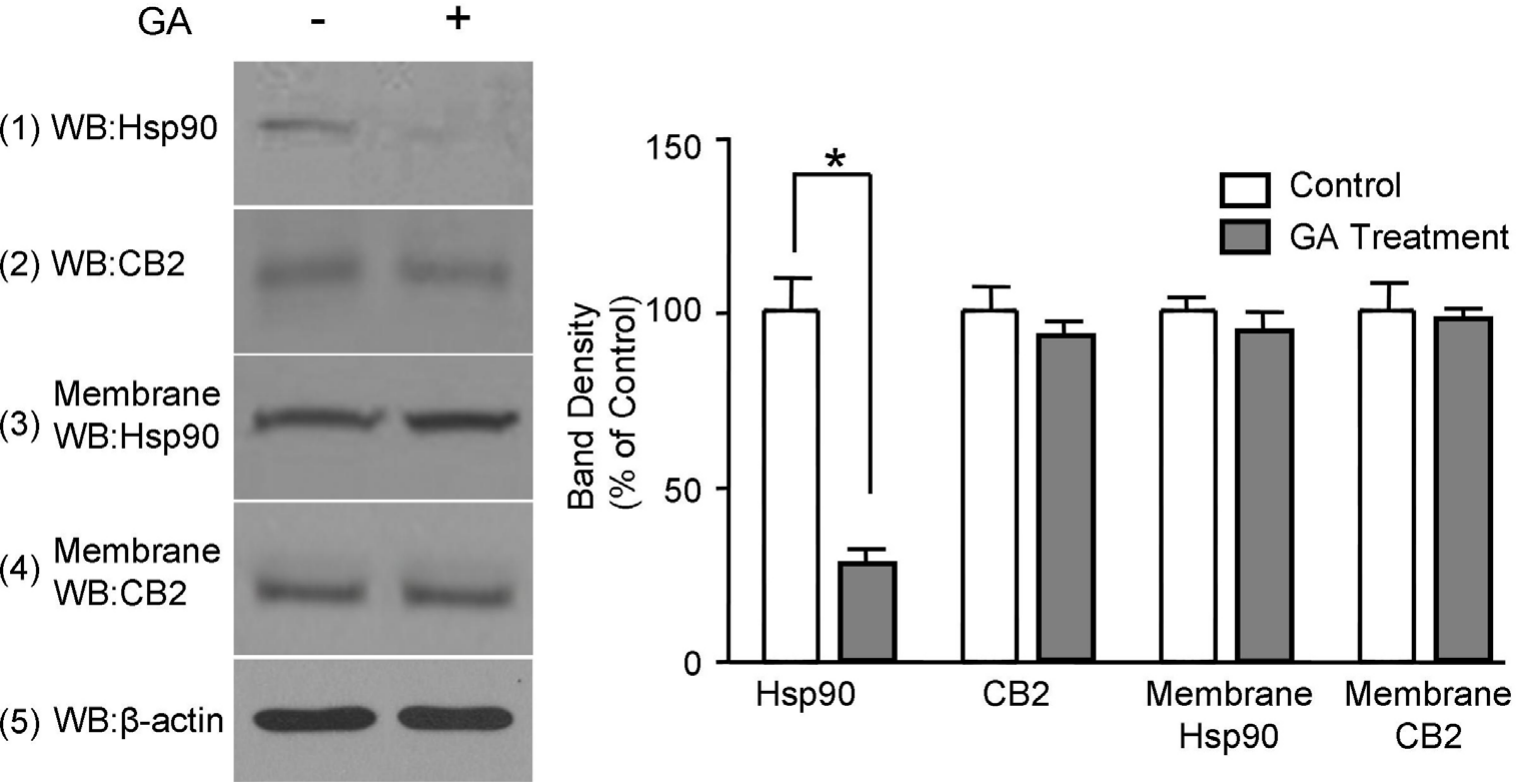
The effect of geldanamycin on the interaction between Hsp90 and the CB2 receptor in trabecular meshwork cells. Intact trabecular meshwork (TM) cells were treated with 10 μM geldanamycin (GA) for 2 h. Membranes were prepared and subjected to coimmunoprecipitation with the anti-CB2 antibody. Then, the samples were immunoblotted with the anti-Hsp90 and anti-CB2 antibodies. The experiment was repeated three times, with similar results as shown in the bar graph. Treatment of TM cells with geldanamycin significantly inhibited the Hsp90 that was coimmunoprecipated by the anti-CB2 antibody.

Geldanamycin, a small molecule that specifically binds to the ATP–binding pocket in Hsp90, but not other chaperone proteins, alters the tertiary structure of Hsp90, and prevents Hsp90 from interacting with target proteins [24]. In this study, the effect on geldanamycin on the interaction between Hsp90 and the CB2 receptor in TM cells was investigated. As shown in Figure 2, geldanamycin treatment did not significantly affect the amount of CB2 receptor and Hsp90 in TM cell membrane preparations. However, treatment with geldanamycin markedly reduced the amount of Hsp90 pulled down by the anti-CB2 antibody in the coimmunoprecipitation experiments. Since geldanamycin specifically inhibits the interaction of Hsp90 with its client proteins, these data provided further evidence for the specific interactions between Hsp90 and the CB2 receptor in TM cells.

### The effects of geldanamycin on CB2 receptor–mediated extracellular signal-regulated kinase 1/2 activation in trabecular meshwork cells

**Figure 3:**
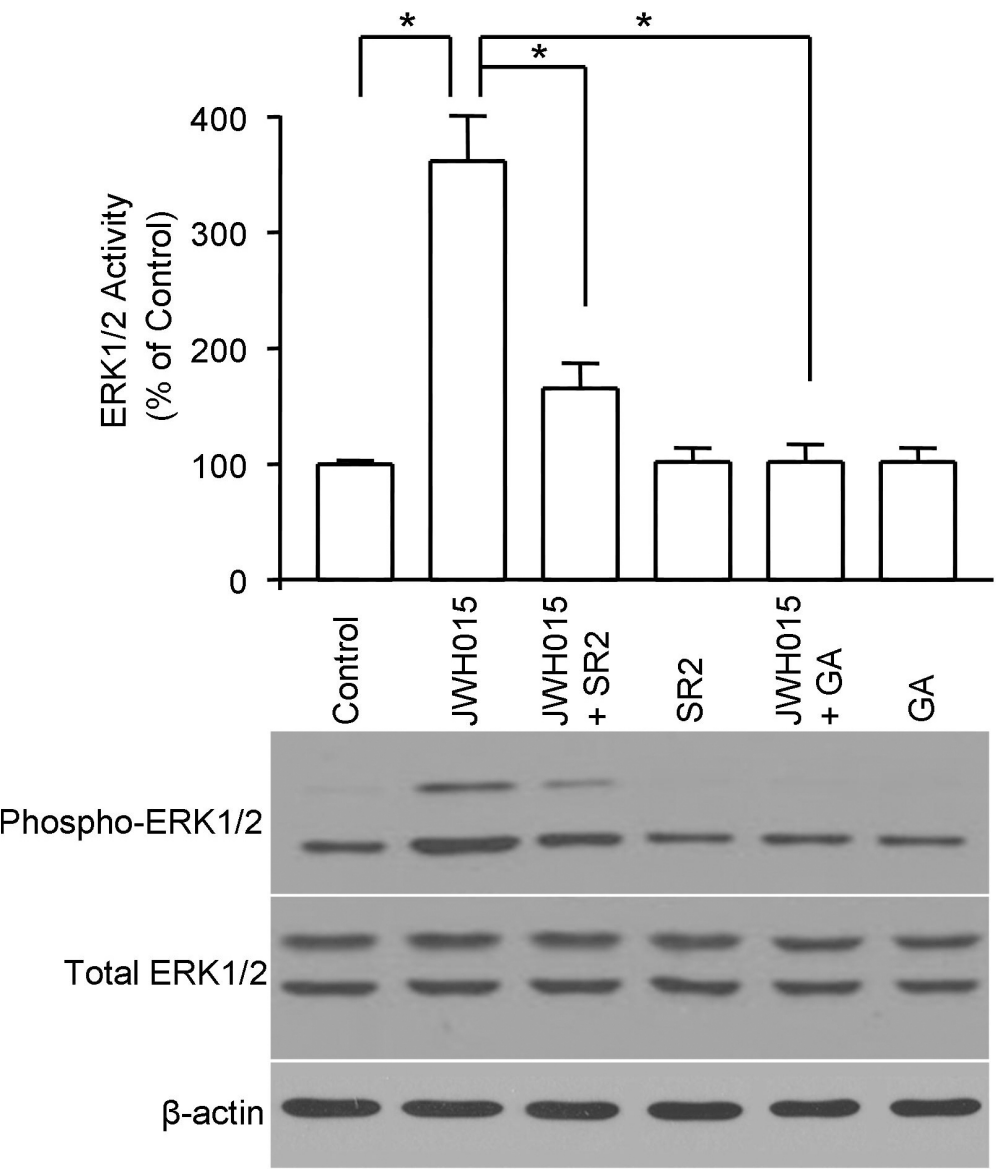
The effects of geldanamycin on CB2 receptor–mediated extracellular-signal-regulated kinases 1/2 (ERK1/2) activation in trabecular meshwork (TM) cells. Serum-deprived intact TM cells were pretreated with 10 μM geldanamycin for 2 h or 1 μM SR144528 (SR2) for 30 min followed by stimulation with 100 nM of JWH 015 for 15 min. Cell lysates were prepared and subjected to western blot analysis using antiphospho-ERK1/2 and total ERK1/2 antibodies. Blots shown are representative of three independent experiments. In the bar graphs, results are normalized to the density of total ERK1/2 bands in the corresponding samples (n=3). * Significant different p<0.05, one-way ANOVA with Newman-Keuls post-test). JWH 015 treatment led to a marked increase in ERK1/2 phosphorylation. SR144528 and geldanamycin significantly inhibited the JWH 015–induced ERK1/2 activation.

CB2 receptor activation leads to the stimulation of the ERK1/2 pathway. Previously, we have shown that JWH 015, a CB2 selective cannabinoid agonist, activates ERK1/2 in TM cells [14]. In this study, we hypothesized that the interaction between the CB2 receptor and Hsp90 might be crucial for CB2 receptor–mediated ERK1/2 activation. To test this hypothesis, geldanamycin was used in the ERK1/2 phosphorylation assay. As shown in Figure 3, treatment of trabecular meshwork cells with 100 nM JWH 015 for 15 min activated ERK1/2, as represented by increased phosphorylation of ERK1/2. This effect of JWH 015 was antagonized by SR144528, a selective CB2 antagonist. Treatment of TM cells for 15 min with geldanamycin by itself had no measurable effect on ERK1/2 phosphorylation. However, pretreatment of TM cells with geldanamycin significantly inhibited JWH 015–induced ERK1/2 phosphorylation. The total ERK1/2 protein levels did not change with all the treatments. These data indicated that through interaction with the CB2 receptor, Hsp90 plays an important role in CB2 receptor–mediated ERK1/2 activation.

### The effects of PD98059 on CB2 receptor–mediated actin cytoskeleton changes in trabecular meshwork cells

**Figure 4:**
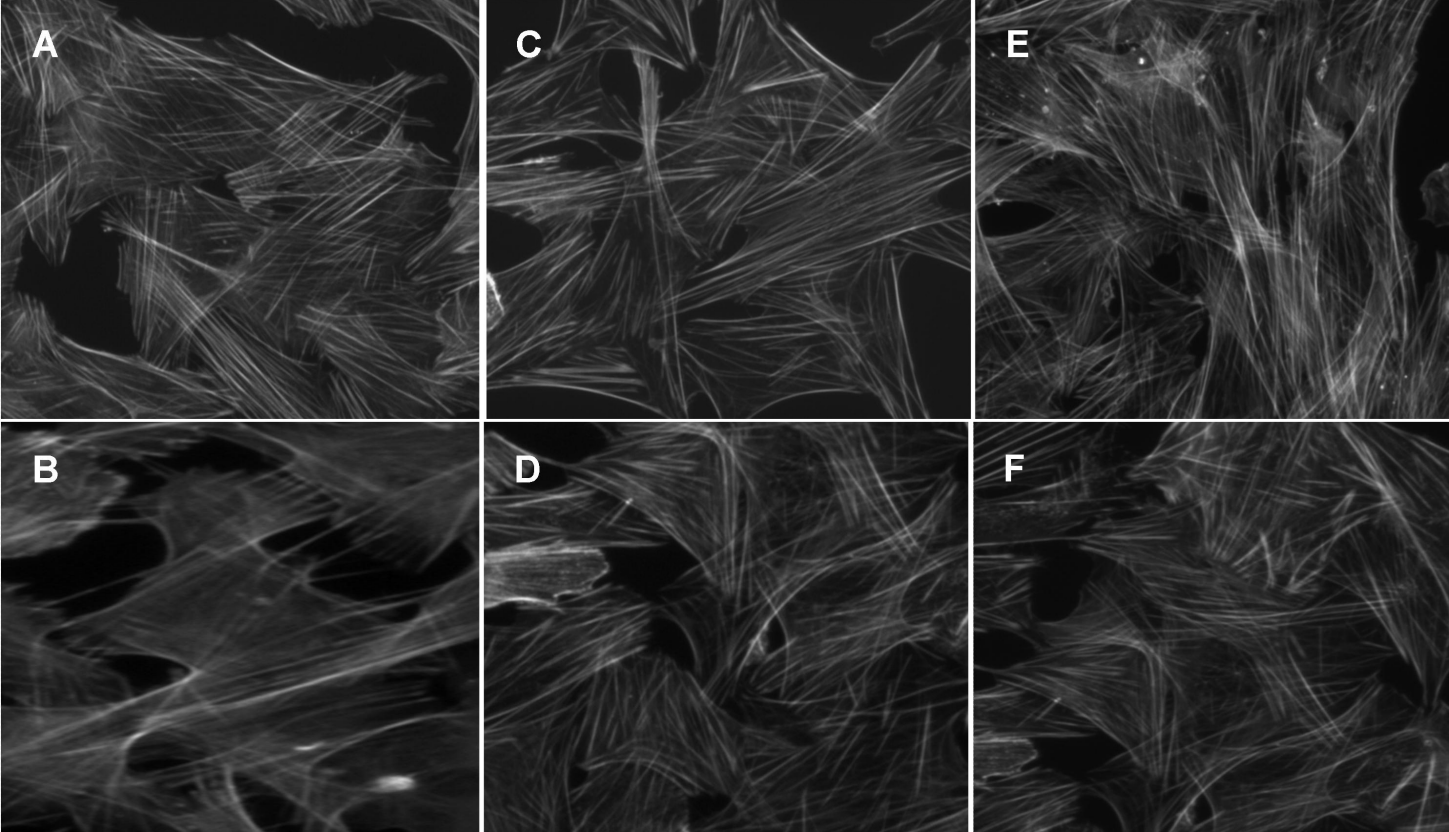
The effects of PD98059 and geldanamycin on CB2 receptor-mediated actin cytoskeleton changes in trabecular meshwork cells. Intact trabecular meshwork (TM) cells were plated on fibronectin-coated (5 μg/ml) coverslips and grown to confluence. Serum-deprived TM cells were pretreated with vehicle (**A** and **B**), 30 μM PD98059 (**C** and **D**) for 30 min and 10 μM geldanamycin (GA; **E** and **F**) for 2 h. Subsequently, the TM cells were treated for 3 h with vehicle (**A**), 100 nM JWH015 (**B**), 30 μM PD98059 (**C**), 100 nM JWH015 plus 30 μM PD98059 (**D**), 10 μM geldanamycin (**E**), and 100 nM JWH015 plus 10 μM geldanamycin (**F**). The TM cells were then fixed with paraformaldehyde and stained with Alexafluor 488-labeled phalloidin as detailed in the Methods section. JWH015 caused a significant decrease in staining for actin stress fibers, compared with vehicle control. PD98059 as well as geldanamycin pretreatment blocked the changes brought about by JWH015.

Previously, we found that treatment of TM cells with the CB2 receptor agonist JWH 015 results in cytoskeleton rearrangement, which can be blocked by the CB2 selective antagonist SR144528 [15]. In addition, we have reported that by acting on CB2 receptors, JWH 015 activates ERK1/2 in TM cells [14]. In the current study, PD98059, an inhibitor of the ERK1/2 pathway, was used to test the hypothesis that ERK1/2 activation is involved in JWH 015–induced actin cytoskeleton rearrangement. As shown in Figure 4A, vehicle-treated (control) TM cells exhibited a well organized actin cytoskeleton with F-actin fibers crossing the body of the cells and forming a dense filamentous network. Cells treated with CB2 agonist JWH 015 showed a significant reduction in the number of stress fibers and a different pattern of these stress fibers, i.e., they are distributed more in the periphery of the cells (Figure 4B). TM cells treated with PD98059 by itself (Figure 4C) exhibited morphology and actin stress fibers similar to that of the control cells. Importantly, as shown in Figure 4D, pretreatment with PD98059 blocked the effects of JWH 015 on the actin cytoskeleton—the cells exhibited most of the cytoskeletal features associated with control cells. These results indicated that ERK1/2 activation plays a key role in the TM cell actin cytoskeleton changes induced by JWH 015.

### The effects of geldanamycin on CB2 receptor–mediated actin cytoskeleton changes in trabecular meshwork cells

Having shown that in TM cells geldanamycin can specifically inhibit the interaction of Hsp90 with CB2 and CB2 receptor–mediated ERK1/2 phosphorylation, we next examined the possible role of Hsp90 in CB2 receptor–mediated actin cytoskeleton changes using geldanamycin. Treatment of TM cells with geldanamycin alone (Figure 4E) had no visible effects on the distribution of actin stress fibers, compared to control TM cells. However, pretreatment of TM cells with geldanamycin for 2 h significantly inhibited JWH 015–induced changes of actin stress fibers, i.e., compared with control group, there was no visible changes in actin stress fiber appearance in the geldanamycin plus JWH 015 group (Figure 4F). Collectively, these data indicated that the interaction of Hsp90 with the CB2 receptor is crucial for CB2 receptor–mediated remodeling of the actin cytoskeleton in TM cells.

## Discussion

The heat shock protein 90 (Hsp90) is a molecular chaperone that is ubiquitously expressed and is responsible for the biogenesis, regulation, and functionality of more than 100 proteins (client proteins) [17–19]. More recently, it has been shown that by forming heterocomplexes with its substrates, Hsp90 plays a key role as a scaffolding protein for various signaling events under non-stress conditions [17–19]. Previously, we identified Hsp90 as a CB2 receptor interacting protein in HEK293 cells transfected with CB2 and in HL-60 cells expressing native CB2, and we established the essential roles of Hsp90 in CB2 receptor–mediated cell migration [20]. In addition, our previous studies demonstrated the existence of functional CB2 cannabinoid receptors in TM cells and activation of CB2 receptors leads to TM cell actin cytoskeleton rearrangement [14,15]. In the current study, we tested the hypothesis that Hsp90 might interact with the CB2 receptor in TM cells and play an important role in CB2 receptor–mediated TM cell signaling and actin cytoskeleton changes.

Using coimmunoprecipitation techniques combined with western blot analyses, in this study we first examined whether there is any interaction between Hsp90 and the CB2 receptor in TM cells. Hsp90 was identified and validated as a constitutive interacting protein of the CB2 receptor in TM cells, because Hsp90 and CB2 could be coimmunoprecipitated by each other in these cells. In addition, neither Hsp90 nor CB2 was immunoprecipitated when a control, non-relevant antibody was used. Thus, we have shown the interaction of these proteins in TM cell lysates in vitro, and this suggests that they may interact in vivo.

Geldanamycin, a small molecule that specifically binds to the ATP–binding pocket in Hsp90, alters the tertiary structure of Hsp90, thus preventing Hsp90 from interacting with target proteins [24]. Geldanamycin has been used frequently as a useful tool to investigate the specificity of interactions of Hsp90 with its client proteins. In this study, pretreatment of intact TM cells with geldanamycin inhibited the interaction of Hsp90 with the CB2 receptor. These results further demonstrate that the interaction between Hsp90 and CB2 is specific in TM cells.

Activation of ERK1/2 activity is one of the well characterized signaling pathways for the CB2 cannabinoid receptor [13]. In previous studies, we have shown that the CB2 selective agonist JWH 015 activates ERK1/2 through the CB2 receptor in TM cells [14]. In the present study, we hypothesized that the specific interaction of the CB2 receptor with Hsp90 might be essential for CB2 receptor–mediated ERK1/2 activation. Our findings from the current study strongly supported this hypothesis, because pretreating intact TM cells with geldanamycin not only disrupted the specific interaction between the CB2 receptor and Hsp90 but also significantly inhibited JWH 015–induced ERK1/2 activation.

Previously, we demonstrated that JWH 015–induced enhancement of aqueous humor outflow facility is mediated through the trabecular meshwork CB2 receptor, with involvement of the ERK1/2 signaling pathway [14]. Furthermore, we found that treatment of TM cells with the CB2 receptor agonist JWH 015 results in cytoskeleton rearrangement, which can be blocked with the CB2 selective antagonist SR144528 [15]. After establishing the specific interaction between Hsp90 and the CB2 receptor, and the importance of this interaction in CB2 receptor–mediated ERK1/2 activation, in the current study we investigated whether disruption of Hsp90-CB2 interaction has an effect on CB2 receptor–mediated actin cytoskeleton rearrangement. We found that pretreating intact TM cells with geldanamycin completely blocks TM cell actin cytoskeleton changes induced by the CB2 agonist JWH 015. These data demonstrate the crucial role of Hsp90–CB2 interaction in the CB2 receptor–mediated actin cytoskeleton rearrangement.

How might Hsp90 play a role in CB2 receptor–mediated ERK1/2 activation and cytoskeleton rearrangement in TM cells? The current study indicates that Hsp90 acts upstream of ERK1/2, since geldanamycin pretreatment blocked JWH 015–induced inactivation of ERK1/2 activation. The current study also demonstrates, for the first time, that pretreatment of TM cells with PD98059, a ERK1/2 pathway inhibitor, blocks JWH 015–induced actin cytoskeleton rearrangement, indicating that ERK1/2 activation plays an important role in CB2 receptor–mediated cytoskeleton changes in TM cells. Previous experimental evidence has suggested Hsp90 is required in activating ERK1/2 induced by various factors or agents [19,25]. Because Hsp90 is a chaperone protein, Hsp90 likely serves as a scaffold to keep the CB2 receptor and its signaling components, including ERK1/2, in close proximity, thus ensuring proper signaling transduction from the CB2 receptor to the actin cytoskeleton.

Overall, the data from this study establish for the first time a specific interaction between Hsp90 and the CB2 receptor in TM cells. This study also provides evidence supporting the notion that ERK1/2 activation is important for CB2-mediated cytoskeleton changes in TM cells. Furthermore, the current study demonstrates that the specific interaction between Hsp90 and the CB2 receptor in TM cells are crucial for CB2 receptor–mediated activation of ERK1/2 and actin cytoskeleton rearrangement. In conclusion, Hsp90 has been identified as an essential molecular chaperone for CB2 receptor–mediated cell signaling and cytoskeleton changes in TM cells.
